# The impact of health literacy on college students’ psychological disturbances and quality of life: a structural equation modeling analysis

**DOI:** 10.1186/s12955-020-01541-7

**Published:** 2020-08-31

**Authors:** Jehad A. Rababah, Mohammed M. Al-Hammouri, Barbara L. Drew

**Affiliations:** 1 Adult Health Nursing Department, Faculty of Nursing, Jordan University of Science and Technology, Irbid, Jordan; 2 Community and Mental Health Department, Faculty of Nursing, Jordan University of Science and Technology, Irbid, Jordan; 3 College of Nursing, Kent State University, Kent, OH USA

**Keywords:** Health literacy, Psychological disturbances, Quality of life, College students, Structural equation modeling

## Abstract

**Background:**

The literature regarding the effect of health literacy on college students’ psychological health and quality of life is scarce. The purpose of conducting this cross-sectional study was to examine the effect of health literacy on certain psychological disturbances (perceived stress, depressive symptoms, and impulsivity) and quality of life of college students.

**Methods:**

A cross-sectional quantitative design was utilized in this study. A total of 310 four-year college students participated in this study. The students completed a demographics questionnaire as well as already established and validated measures of health literacy, perceived stress, depressive symptoms, impulsivity, and quality of life. Structural equation modeling was performed to analyze the data to explore the effect of health literacy on the psychological disturbances and quality of life.

**Results:**

The results showed that health literacy has a negative effect on three psychological disturbances commonly experienced by college students; perceived stress, depressive symptoms, and impulsivity. In addition, the effect of health literacy on the quality of life was positive.

**Conclusion:**

The proposed conceptual model was supported. College students’ counseling staff could use the findings to better address students’ needs pertinent to psychological health and quality of life. Future research is warranted to develop a more comprehensive model that explains the role of health literacy in determining college students’ psychological health and quality of life.

## Background

Health literacy is a concept that describes the ability to acquire, understand, process, and utilize health-related information [1–3]. Individuals could be classified as having adequate/good or limited/poor health literacy levels. Being health literate enables the person to make informed decisions regarding his/her own health or the health of others, family members for example [1, 3, 4]. Conversely, limited health literacy negatively affects the overall health status of individuals and communities [5]. Despite its importance in determining the overall health and well-being, health literacy remains an area of inquiry that is often neglected in research [3]. One of the main existing issues is that most health literacy research studies have been conducted in the U.S., Europe, and Australia. Another concern is the focus on measuring functional health literacy and the reading abilities [1, 3]. Moreover, Nutbeam and colleagues maintained that the multidimensional nature of health literacy makes it a challenging concept to define and measure [2].

The relationship between health literacy and overall health has recently gained more attention in research, and limited health literacy is reportedly a key determinant of health and well-being. Health promotion and disease prevention are negatively affected by limited health literacy [3, 6]. In addition, limited health literacy affects a variety of health aspects including management of chronic health conditions, making decisions regarding healthy habits, misunderstanding of and poor compliance with prescribed medications, more frequent hospital admissions, and mortality [3, 5]. Moreover, utilization of expensive healthcare services including specialized and emergency services increases with limited health literacy [7, 8]. Such detrimental effects increase the burden on healthcare systems and inflate costs [7, 9].

Regarding the health literacy of college students, a review of the literature showed that research of this area is still evolving. A focus point of investigation has been the measurement of the level of health literacy among college students. Sansom-Daly and colleague conducted a systematic review and found that at least 60% of adolescents and young adults, including college students, have adequate health literacy [10]. Other research studies reported lower levels of health literacy [11, 12]. In addition, disparities of health literacy in college students exist related to age, gender, and field of study [13, 14]. In terms of its relationship with college students’ health, lower health literacy was associated with such health indicators of physical health as obesity and smoking [10]. However, evidence regarding the association between health literacy and other health domains including psychological disturbances and quality of life (QOL) is limited. The following discussion will summarize the literature regarding college students’ psychological disturbances and QOL.

### Psychological disturbances among college students

College students experience a variety of psychological disturbances throughout their study journey. Compared to their pre-enrollment status, college students reported worsening of psychological well-being during their course of study [15]. The level of distress experienced by college students is higher than the level reported by the general population [15, 16]. Perceived stress positively correlates with psychological distress and is frequently reported as a main psychological disturbance experienced by college students [17]. Previous studies showed that perceived stress negatively impacts academic performance as well as physical and psychological health [18, 19].

Another major concern regarding college students’ psychological health is depressive symptoms. Depressive symptoms affect college students around the globe [20, 21]. The prevalence of depressive symptoms is reportedly higher in college students than in the general population [22]. Depressive symptoms are not only associated with poor academic performance [23], but also convey detrimental effects on college students’ health including an increased risk of alcohol use [24], unhealthy eating [25], and sleep disturbances [26]. Moreover, there is evidence that suicidal ideation is a consequence of even mild to moderate levels of depressive symptoms among college students [27].

College students respond to psychological distress in diverse ways. Impulsivity, defined as “a predisposition toward rapid, unplanned reactions to internal or external stimuli without regard to the negative consequences of these reactions to the impulsive individual or to others” [28], is considered a key determinant of an individual’s behaviors and reaction to distress. In general, impulsivity affects nearly all aspects of daily life [29]. Many research studies have been conducted to better understand the relationship between impulsivity and unhealthy behaviors. According to the literature, impulsivity is associated with increased risk of problematic drinking and unhealthy eating [30, 31]. Chamorro and colleagues maintained that the impact of impulsivity on health behaviors is usually more apparent among younger populations, e.g. college students [32]. In addition, impulsivity interacts with psychiatric problems such as post-traumatic stress disorder (PSTD) and suicidal ideation and attempts [33, 34].

### QOL among college students

While there is no consensus on the definition of QOL, many resources agree that it involves an individual’s satisfaction with health and life [35, 36]. Physical and human resources as well as student services affect college students’ QOL [37]. Healthy populations, like most college students, have better QOL compared to patients with chronic diseases [38]. However, the literature shows that both psychological and physical health can impact college students’ QOL [39]. In turn, QOL is correlated with academic achievement and health promotion of college students [37]. However, the existing literature regarding health literacy effect on college students’ QOL is still not well-understood [36]. Thus, this study was conducted to examine the effect of health literacy on certain psychological disturbances (perceived stress, depressive symptoms, and impulsivity) and quality of life of college students.

Based on the previous discussion, college students are at high risk for different psychological disturbances including depressive symptoms, stress, and impulsivity. Such disturbances could mitigate college students’ QOL. As presented earlier, the health literacy of many college students is limited. However, the effect of health literacy on college students’ psychological health and QOL is still not well-understood. Therefore, this study was conducted to better understand how health literacy affects certain psychological disturbances experienced by college students and their QOL. The current study was guided by a conceptual model that has been developed based on the extensive review of the literature.

Health literacy is hypothesized in this model as the latent exogenous construct. Health literacy was determined using the nine scales of the health literacy questionnaire (HLQ) [40]. Psychological status and QOL were proposed as the latent endogenous variables. Three psychological disturbances (perceived stress, depressive symptoms, and impulsivity) were purported as key determinants of college students’ psychological status. QOL was determined using the four domains of health and functioning, social and economic, psychological/spiritual, and family [35]. Two research hypotheses were formulated to test the model proposed in this study:

Hypothesis #1: the greater college students’ health literacy, the less psychological disturbances.

Hypothesis #2: college students’ health literacy positively affects their QOL.

## Methods

### Design and setting

This study was conducted using a cross-sectional quantitative design. It was carried out at a large, public university in north Jordan.

### Sampling and participants

Proportional quota sampling method was used to conduct the current study. This sampling method was employed to recruit participants representative of both the different fields of study and the year of study. Undergraduate college students were invited to participate in the study. A total of 310 undergraduate students completed the data collection questionnaires. The average age of study participants was 20.89 years old (SD = 2.0). Male students represented 52.9% of participants. The average BMI was 24.3 kg/m^2^ (SD = 4.45) with a range from 16.22 to 44.4 kg/m^2^. Table 1 summarizes the demographics of the participants.

**Table 1:** Participants’ Demographic Characteristics

Variable	Total (*N* = 310)	
	N	Percentage
Gender		
Male	164	52.9
Female	146	47.1
Year of Study		
First	65	21
Second	78	25.2
Third	55	17.7
≥ Fourth	112	36.1
Current Smoking		
Yes	97	31.3
No	213	68.7
Field of Study		
Health-related^a^	204	65.8
Other^b^	106	34.2

^a^Health-related fields included Medicine, Dentistry, Pharmacy, Nursing, and Applied Medical Sciences

^b^Other included Engineering, Agriculture, Veterinary Medicine, General Sciences, Computer Sciences, and Architecture

### Data collection

The participants were invited to complete paper-based questionnaires including a demographics questionnaire, HLQ, Perceived Stress Scale-10 (PSS-10), the Center for Epidemiologic Studies Depression Scale-Revised (CESD-R), Barret Impulsiveness Scale (BIS-11) (BIS-11), and the generic version of the Quality of Life Index (QLI). College students’ height and weight were obtained by one trained research assistant using the same scale to enhance reliability of measurements. Using the obtained height and weight, the BMI was calculated as the college student’s weight (in Kilograms) divided by the square of height (in meters).

#### Health literacy

The HLQ is composed of 44 items classified under nine distinct scales, identified earlier, representing the health challenges and needs of people [40]. The nine scales of the HLQ are: a) “Feeling understood and supported by healthcare providers”, b) “Having sufficient information to manage my health”, c) “Actively managing my health”, d) “Social support for health”, e) “Appraisal of health information”, f) “Ability to actively engage with healthcare providers”, g) “Navigating the healthcare system”, h) “Ability to find good health information”, and i) “Understand health information”. The first five scales contain items with responses ranging from one (strongly disagree) to four (strongly agree). On the other hand, the scales six through nine include items with five response ranging from one (cannot do or always difficult) to five (always easy). It is recommended to obtain the scores of the nine scales instead of calculating a total score of the HLQ [40]. To obtain the scores of the nine scales, the average of the items is obtained. Possible total scores range from one to four in the first five scales and one to five in the scales six through nine. Higher scores of the HLQ scales indicate better levels of health literacy. Internal consistency of the nine scales of the HLQ was supported in the current study with Cronbach’s α values ranging from .71 to .83.

#### Perceived stress

Perceived stress was measured using the PSS-10; a 10-item, easy to understand tool [41]. The participants were asked to report their own appraisal regarding how much specific situations have been stressful during the last month. The PSS-10 employs a five-point Likert scale ranging from zero (never) to four (very often). Positive items were reversed before running analyses and then the total score was obtained by summing up the scores of individual items. The higher the total score on the PSS-10, the greater the perceived stress is. The Cronbach’s α value for the PSS-10 in this study was .73.

#### Depressive symptoms

The CESD-R was used to screen for depressive symptoms among the participants [42]. The CESD-R is a 20-item instrument that includes questions about the nine groups of depressive symptomatology as described by the Diagnostic and Statistical Manual of Mental Disorders, fifth edition (DSM-V). Each item is self-reported on a scale from zero (not at all or less than 1 day) to four (nearly every day for 2 weeks). The total score is calculated as the sum scores of individual items of the CESD-R. The higher the CESD-R score, the more severe depressive symptoms are. The Cronbach’s α value for the CESD-R in this study was .93.

#### Impulsivity

The BIS-11 was used to measure participants’ impulsivity. The BIS-11 is a 30-item scale that could be self-rated on a four-point scale ranging from one (rarely/never) to four (almost always/always) [43]. The scale is composed of six, first order factors: attention, cognitive instability, motor, perseverance, self-control, and cognitive complexity. After reversing the negatively worded items, the total impulsivity score is calculated by summing up the item scores. According to Patton and colleagues, higher scores indicate higher level of impulsivity. The Cronbach’s α value for the BIS-11 in this study was .66.

#### QOL

The generic version of the QLI was administered to measure participants’ QOL [35]. Besides measuring the overall QOL, the QLI provides information regarding the QOL in the following domains: a) health and functioning, b) social and economic, c) psychological/spiritual, and d) family. The QLI involves two sections with a total of 66 items: the first section is used to assess satisfaction with 33 items, and the second assesses the importance of those 33 items to the individual. A six-point scale is used in the QLI, with a score of one means “very dissatisfied” and “very unimportant” and a score of six means “very satisfied” and “very important”. The total QLI score ranges from zero to 30 following a syntax provided by the instrument developers, with a higher score indicating better QOL. The Cronbach’s α value for the QLI in this study was .91.

### Data analysis

In this study, SPSS (Version 23) and AMOS (Version 23) were used to perform the analyses. SPSS was used to perform descriptive analysis and estimate the internal consistency of the instruments. Structural Equation Modeling (SEM) was performed using AMOS. SEM, with Maximum Likelihood Estimation, was conducted and the process involved two main phases. In the first phase, a pooled Confirmatory Factor Analysis (CFA) was done to assess the appropriateness of the proposed measurement model prior to going forward into the second phase. The main purpose of performing CFA was to assess the validity and other parameters regarding the factor structure of the measurement model. In the pooled CFA, all latent constructs were entered into the analysis and tested simultaneously. The specific parameters and criteria used to assess the measurement model appropriateness are discussed in the results section. It is worth noting that the pooled CFA method was used for two reasons: a) it is more useful to assess the whole measurement model instead of examining the factor structure of individual constructs (e.g. health literacy), and b) presenting the results regarding the CFA for each construct is beyond the limit of a single research article.

In the second phase, the structural model (i.e. SEM model causal pathways) was tested. Initially, the direct effect of health literacy on the psychological disturbances (hypothesis 1) and QOL (hypothesis 2) was examined. In this study, the results were not interpreted solely based on the Chi Square statistic because it is sensitive to large sample size [44]. According to Hair and colleagues, model fitness is better assessed using at least one fit index from three categories of model fitness (i.e. absolute fit, incremental fit, and parsimonious fit) [44]. Thus, the following indices were set a priori and used in the analyses presentation and interpretation: a) absolute fit: Root Mean Square of Error Approximation (RMSEA) < .07, b) incremental fit: Comparative Fit Index (CFI) > .92, and c) parsimonious fit: Chi Square/Degrees of Freedom (Chisq/df) < 5. RMSEA and CFI values were set at these levels considering the number of observed variables in the conceptual model and the sample size [44]. Standardized regression weights (β) were also assessed and interpreted during the second phase of analysis.

## Results

### Preliminary analysis: measurement model using pooled CFA

The factor structure of the measurement model was assessed first. Discriminant validity was initially evaluated based on the correlations among the constructs of the proposed model. “Low correlations” are indicative of discriminant validity [44]. The results showed that the correlations were all moderate (ranging from −.46 to −.63); which could be a threat to the discriminant validity. To further assess the discriminant validity, the values of the squared root of the average variance extracted (AVE) for each construct were calculated and compared to the inter-construct correlations. Discriminant validity is supported when the squared root AVE of a construct is greater than the inter-construct correlation with any other construct in the model [44]. The results (see Table 2) supported the discriminant validity with values of squared root AVE exceeding the inter-construct correlations, except for the construct psychological disturbances. On the other hand, convergent validity of the individual constructs was evaluated based on the AVE of ≥ .5 [44]. Then composite reliability (CR) of the individual constructs were calculated to assess factor reliability. All CR values of the constructs, except the construct “psychological disturbances, were above the cutoff point of .7 suggested by Hair et al.; indicating that convergent validity or internal consistency is supported. In Table 2, inter-construct correlations are summarized along with the AVE and CR for each construct.

**Table 2:** Reliability and Validity of The Measurement Model

	HL	Psychological Disturbances	QOL	CR	AVE
HL	**.74**			.91	.54
Psychological Disturbances	−0.46	**.61**		.63	.37
QOL	0.59	−0.63	**.80**	.87	.63

*HL* Health literacy, *QOL* Quality of life, *CR* Composite reliability, *AVE* average variance extracted

Note: bold diagonals are squared root of AVE, off-diagonals are correlations

**Fig. 1:**
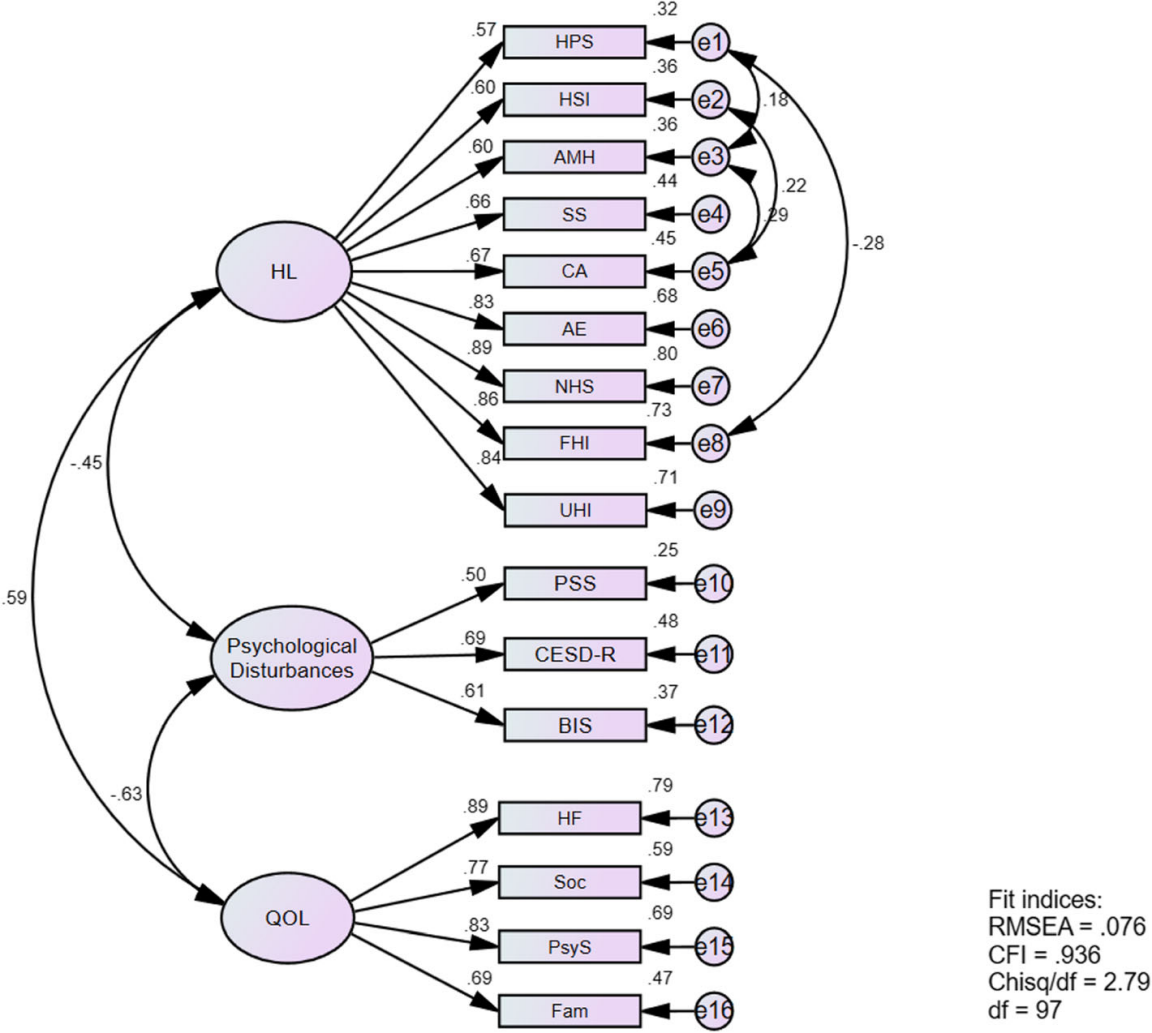
Summary of Measurement Model Fitness, Reliability, and Validity. HL: Health Literacy, HPS: Feeling understood and supported by healthcare providers, HIS: Having sufficient information to manage my health, AMH: Actively managing my health, SS: Social support for health, CA: Appraisal of health information, AE: Ability to actively engage with healthcare providers, NHS: Navigating the healthcare system, FHI: Ability to find good health information, UHI: Understand health information, PSS: Perceived Stress Scale-10, CESD-R: Center for Epidemiologic Studies Depression Scale-Revised, BIS: Barret Impulsiveness Scale-11, QOL: Quality of Life, HF: Health and Functioning, Soc: Social and Economic, PsyS: Psychological/Spiritual, Fam: Family.

Regarding the fit indices, the measurement model showed less than acceptable fit with RMSEA = .09, CFI = .904, and Chisq/df = 3.58. An investigation of the modification indices revealed that the fit of the measurement model could be improved through freeing some within-factor paths (namely *e3-e5, e2-e5, e1-e8, and e1-e3*). The model fitness indices have improved after adding covariances between these paths; RMSEA = .076, CFI = .936, and Chisq/df = 2.79 (See Fig. 1). Based on the results of the measurement model fit as well as its reliability and validity, we moved forward with conducting the SEM. Figure 1 shows the measurement model and the results pertinent to its reliability and validity including the standardized factor loadings and R^2^.

### Hypotheses testing

**Fig. 2:**
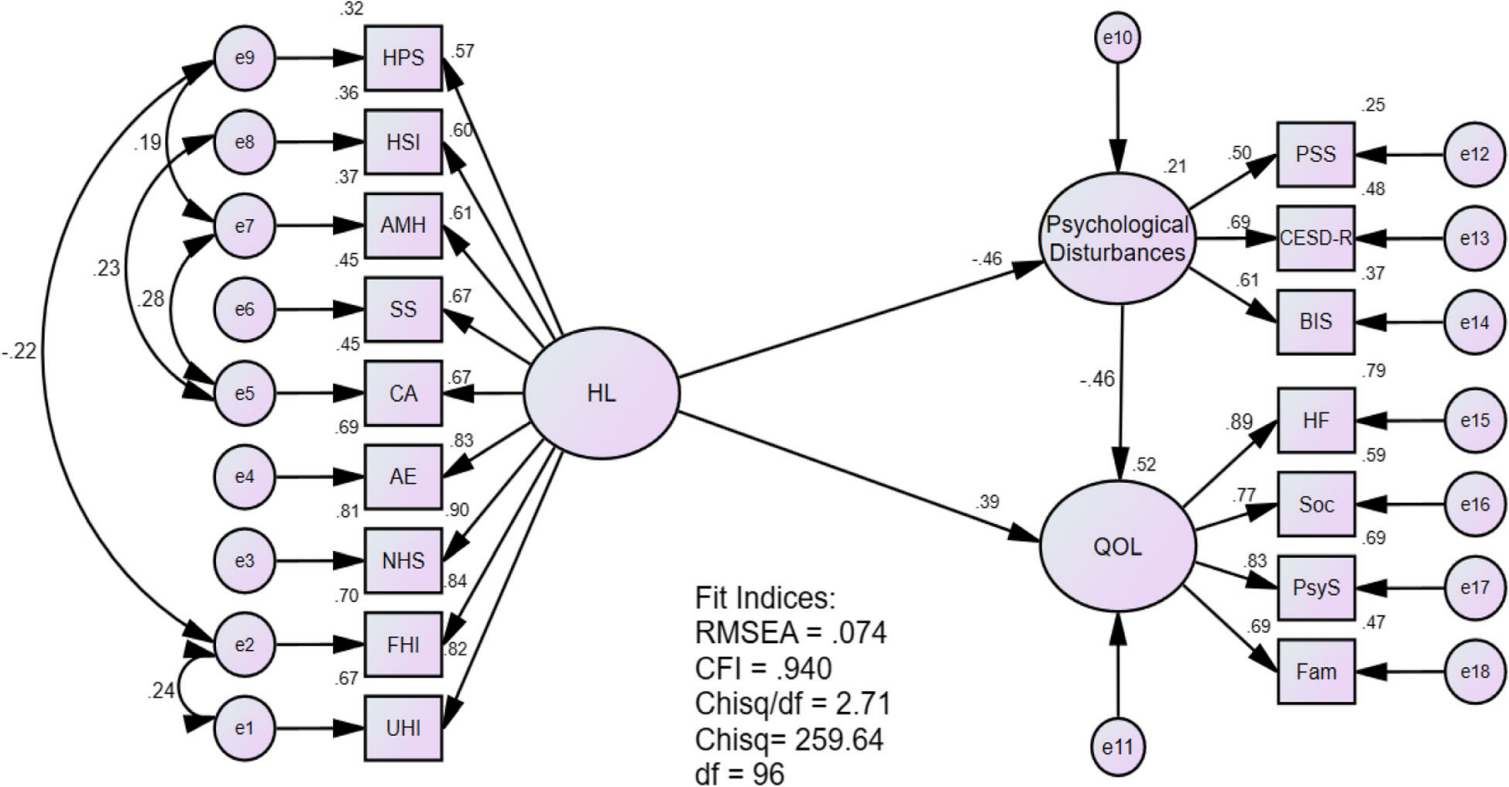
SEM Model. HL: Health Literacy, HPS: Feeling understood and supported by healthcare providers, HIS: Having sufficient information to manage my health, AMH: Actively managing my health, SS: Social support for health, CA: Appraisal of health information, AE: Ability to actively engage with healthcare providers, NHS: Navigating the healthcare system, FHI: Ability to find good health information, UHI: Understand health information, PSS: Perceived Stress Scale-10, CESD-R: Center for Epidemiologic Studies Depression Scale-Revised, BIS: Barret Impulsiveness Scale-11, QOL: Quality of Life, HF: Health and Functioning, Soc: Social and Economic, PsyS: Psychological/Spiritual, Fam: Family.

The SEM analysis revealed the following fit indices: RMSEA = .074, CFI = .94, Chisq/df = 2.71 (df = 96). These fit indices, except for the RMSEA, meet the criteria set a priori. The model is depicted with a summary of model fit indices and the other parameters (see Fig. 2).

#### Hypothesis #1

Regarding Hypothesis #1, the R^2^ value of the psychological disturbances construct was .21; meaning that health literacy explains 21% of the predicted variance. The standardized regression weight value regarding the effect of health literacy on the psychological disturbances construct was statistically significant (β *=* −.46, *p* < .001). This means that one standard deviation increase in health literacy produces a .46 standard deviation reduction in the psychological disturbances (Table 3).

**Table 3:** Standardized Regression Weights (β) and Interpretation of The Hypotheses

Hypothesis	β	S.E	C.R	Interpretation
H1: Psychological Disturbances ←HL	−.46**	.46	− 5.25	When HL goes up by 1 standard deviation, Psychological Disturbances goes down by 0.46 standard deviations.
H2: QOL ←HL	.37**	.45	5.87	When HL goes up by 1 standard deviation, QOL goes up by 0.37 standard deviations.

** *p* < .001

*H1–2* Hypothesis 1–2, *HL* Health Literacy, *QOL* Quality of Life, *S.E* Standard Error, *C.R* Critical Ratio

Note: The S. E and C. R values reported in this table are the unstandardized estimates

#### Hypothesis #2

On the other hand, the R^2^ value of the QOL construct was .52; indicating that health literacy explains 52% of the predicted variance. The β value regarding the effect of health literacy on QOL was also statistically significant (β = .37, *p* < .001). In other words, an increase of one standard deviation in the health literacy construct results in .37 standard deviation increase in the QOL construct. These results (see Table 3) indicate that both hypotheses were supported; the greater college students’ health literacy, the less psychological disturbances and the better QOL.

## Discussion

Health literacy plays a key role in determining the overall health status of individuals and communities. Research on health literacy is still evolving and the effect of health literacy on college students’ psychological health and QOL is not thoroughly understood. Therefore, the authors intended to investigate the role of health literacy on these domains of health in this study. A model was proposed, and two distinct hypotheses were tested using SEM analyses. The measurement model was assessed, and the results indicated that its factor structure is adequate. The structural model was then assessed, and the results indicated that the model fitness indices are supported. Results regarding the structural model indicated that the proposed hypotheses were supported. Health literacy had both a statistically significant negative effect on the psychological disturbances under investigation and a statistically significant positive effect on college students’ QOL.

Perceived stress, depressive symptoms, and impulsivity are among the many psychological disturbances often experienced by college students. The literature shows that such disturbances negatively affect college students’ overall health and academic performance [29, 33]. In addition, psychological disturbances negatively influence health literacy and help-seeking behaviors of college students [45]. However, the opposite effect of health literacy on psychological disturbances, has not been studied yet, to the best of the authors’ knowledge. Thus, the current study adds to the existing knowledge that health literacy could decrease the risk of common psychological problems. In other words, improving college students’ health literacy could lead to better outcomes evidenced by a reduction in the perceived stress, depressive symptoms, and impulsivity.

Regarding the effect of health literacy on college students’ QOL, a recent systematic review and meta-analysis showed that health literacy has a mild correlation with college students’ QOL [36]. Further investigation of this area was recommended by Zheng and colleagues [36]. Support of the second hypothesis in this study is consistent with the findings of that systematic review. The current study did not include an investigation of the relationship between health literacy, QOL, and academic performance. However, previous research showed that improving college students’ QOL could play an essential role in achieving better academic and health-promoting outcomes [37]. Hence, the results of the current study open the door for further investigation regarding the improvement of college students’ academic performance through enhancement of health literacy and QOL.

### Implications

The results of this study could be useful for college staff involved with counseling services provided to college students. Schwitzer and colleagues reported that seeking counseling services is associated with positive improvement of academic performance [46]. It was, also, concluded in a systematic review that expanding the expertise of those involved with college students’ counseling has a crucial impact on supporting college students [47]. Thus, understanding how health literacy affects psychological well-being and QOL could be useful for university counseling staff. Offering training for counseling personnel regarding health literacy and its effects on college students’ psychological disturbances and QOL is paramount. It should be emphasized that providing valid information to college students could improve their psychological well-being and QOL. In addition, the results of this study could be useful for college students to increase their awareness regarding how health literacy affects their psychological health and QOL.

Healthcare workers interested in college students’ health and health promotion could also benefit from the results of this study. Health literacy, as mentioned earlier, enables individuals to make appropriate, informed decisions regarding their own health and the health of others. Enabling individuals to make such decisions lies at the core of providing patient-centered care; a fundamental element of providing quality health care. Thus, understanding how health literacy affects psychological well-being and QOL needs to be acknowledged by healthcare professionals involved with promoting college students’ health. Healthcare providers are encouraged to meet college students’ needs through designing and implementing health promotion programs that emphasize the key role of informing and enabling individual college students. In turn, this could carry a positive effect on adopting a healthy lifestyle that could minimize the risk for chronic health problems.

### Recommendations for future research

The current study provides a preliminary model about the effect of health literacy on the psychological disturbances and QOL of college students. Future research, both exploratory and interventional, is needed to better understand this area of research. Further research that integrates other indicators of psychological stress, such as anxiety, is warranted. Incorporating other domains of health, such as physical health and health promotion, seems necessary to provide more insights regarding the potential effect of health literacy on college students’ overall health. We employed a SEM analysis and examined the impact of health literacy on the psychological disturbances and QOL of college students. However, there is a need to expand the model and integrate potential mediators and/or moderators that should also be investigated. Examples include college students’ age, gender, and field of study. For example, the literature suggests that college students’ gender is considered as a source of disparity of health literacy [13, 14]. Conducting multisite research with college students from different cultural and sociodemographic backgrounds should also be considered.

### Limitations

The results of the current study should be interpreted within the context of the study limitations. This study was conducted at one setting; consequently, the generalizability of the findings might be limited. A cross-sectional data collection approach was used in the current study. Conducting a longitudinal research with data collection at different time points, for example at the end of each academic year, would add more rigor. Regarding sampling, a proportional quota sampling method was used. However, the descriptive statistics showed that we had more college students from the health-related faculties, like medicine and nursing. The literature shows that the number of health education topics studied is positively correlated with college students’ health literacy [12].

## Conclusions

The results of this study showed that health literacy mitigates certain psychological disturbances; perceived stress, depressive symptoms, and impulsivity. In addition, it has a positive effect on improving college students’ QOL. These results add insights to the existing literature and provide ideas regarding conducting future research. The conceptual model was supported by the results; however, other domains of health should be added and tested. Expanding the model could play a key role in strengthening health promotion programs designed for college students.

## Data Availability

Per the regulations of the host university, sharing the datasets used and analyzed in the current study is not permissible.
